# CRISPR-Cas9-guided amplification-free genomic diagnosis for familial hypercholesterolemia using nanopore sequencing

**DOI:** 10.1371/journal.pone.0297231

**Published:** 2024-03-20

**Authors:** Sijia Xu, Hiroki Shiomi, Yugo Yamashita, Satoshi Koyama, Takahiro Horie, Osamu Baba, Masahiro Kimura, Yasuhiro Nakashima, Naoya Sowa, Koji Hasegawa, Ayako Suzuki, Yutaka Suzuki, Takeshi Kimura, Koh Ono

**Affiliations:** 1 Department of Cardiovascular Medicine, Graduate School of Medicine, Kyoto University, Kyoto, Japan; 2 Division of Translational Research, National Hospital Organization, Kyoto Medical Center, Kyoto, Japan; 3 Department of Computational Biology and Medical Sciences, Graduate School of Frontier, Tokyo University, Tokyo, Japan; Odense University Hospital, DENMARK

## Abstract

Familial hypercholesterolemia is an inherited disorder that remains underdiagnosed. Conventional genetic testing methods such as next-generation sequencing (NGS) or target PCR are based on the amplification process. Due to the efficiency limits of polymerase and ligase enzymes, these methods usually target short regions and do not detect large mutations straightforwardly. This study combined the long-read nanopore sequencing and CRISPR-Cas9 system to sequence the target DNA molecules without amplification. We originally designed and optimized the CRISPR-RNA panel to target the low-density lipoprotein receptor gene (*LDLR*) and proprotein convertase subtilisin/kexin type 9 gene (*PCSK9*) from human genomic DNA followed by nanopore sequencing. The average coverages for *LDLR* and *PCSK9* were 106× and 420×, versus 1.2× for the background genome. Among them, continuous reads were 52x and 307x, respectively, and spanned the entire length of *LDLR* and *PCSK9*. We identified pathogenic mutations in both coding and splicing donor regions in *LDLR*. We also detected an 11,029 bp large deletion in another case. Furthermore, using continuous long reads generated from the benchmark experiment, we demonstrated how a false-positive 670 bp deletion caused by PCR amplification errors was easily eliminated.

## Introduction

Familial hypercholesterolemia (FH) is an autosomal hereditary disorder that is linked to impaired low-density lipoprotein (LDL)-particle clearance that accelerates coronary artery disease (CAD) and leads to premature death.

FH remains underdiagnosed and undertreated globally. Most countries have a diagnostic rate of less than 1% [1]. Although homozygous FH presenting early clinical symptoms is rare (≤ 1:1000,000), heterozygous FH is estimated to be frequent (~1:300). However, it is often unnoticed until individuals reach their forties [2, 3]. At the time of the registry, about 40% of patients with FH were not on lipid-lowering therapy [3]. A criteria-based diagnosis like the Dutch Lipid Clinical Network (DLCN) uses a scoring system comprising multiple elements such as untreated LDL cholesterol (LDL-C) levels and family history. However, these data are not always available. Also, physical sign evaluation may be affected by selection bias. These factors increase the uncertainty of FH diagnosis.

Identification of pathogenic mutations provides a definite FH diagnosis. More than 2,100 pathogenic (or likely pathogenic) mutations in the LDL receptor gene (*LDLR*) have been collected in the ClinVar database (https://www.ncbi.nlm.nih.gov/clinvar). Mutations in proprotein convertase subtilisin/kexin type 9 (*PCSK9*) and apolipoprotein B (*APOB*) may also cause FH, but their presence is not as common as *LDLR*.

FH mutations have been considered independent CAD risk factors [4]. Among patients with severe hypercholesterolemia (LDL-C ≥ 190 mg/dL), mutation carriers have three times the risk of developing CAD [5], partly due to the tendency of a lifelong increase in the arterial deposits of LDL-C.

FH genetic testing has relied on amplification-based methods, such as next-generation sequencing (NGS) or PCR, for a long time. Due to the efficiency limits of polymerase and ligase enzymes, NGS generates short reads (usually ~150–250 bps). Because of this, it is commonly used to detect small variants in protein-coding regions [6]. Among patients classified as having "possible FH" by the Dutch lipid clinical network criteria, NGS exon sequencing reported that the FH-mutation detection yield was about 15% [7]. FH mutations are more diverse than expected. Mutations located in non-protein-coding regions, such as promoters, splicing regions, or deep introns, can also give rise to FH [8–10].

Moreover, it is difficult for PCR methods to detect large insertions/deletions (Indels). Long-range PCR enzymes can amplify up to 40 kb of genomic DNA (gDNA) [11], but primer design and reaction remain challenging for highly repetitive and GC-rich regions, such as the promoter region and exons 4, 7–9, and 13 of *LDLR*. Each amplicon requires specific reaction conditions for the PCR, increasing the labor requirements and level of bias. Furthermore, it is known that epigenetic factors, such as the methylation of regulatory elements, also frequently alter the expression of genes [12]. However, these features are entirely lost during amplification processes.

Long-read sequencing technologies have brought new solutions to the blind points of traditional genetic testing in recent years. The Oxford Nanopore Technology (ONT) sequencing system can generate long reads of dozens of kilobases, and detecting mutations is enabled for long targets. The platform also has sequencing protocols for PCR amplicons, but this does not take full advantage of long-read sequencing.

The CRISPR-Cas9 system, directed by a customized CRISPR ribonucleic acid (crRNA), can bind and cut double-strand DNA in a region of interest (ROI) [13, 14], which inspires an amplification-free target sequencing. The attachment of nanopore sequencing adapters requires 5’-phosphates of DNA molecules. First, the existing ends of gDNA molecules are dephosphorylated. After that, Cas9-guided cleavage on flanking sites of ROIs exposes new phosphate groups at the cutting sites. Sequencing adapters can now be added. As a result, ROIs are targeted and sequenced directly without amplification steps. This strategy can bypass the genomic complexity within the ROIs. The approach is officially suggested to enrich a single ROI < 20 kb, partly because long molecules are more easily broken during the experiment. The resulting coverage has a decreasing tendency toward the middle of long ROIs. This strategy has been used in analyzing cancer susceptibility and optimizing the genomic alignment in plants [15–17]. There are no related workflows to identify FH.

Our study successfully applied the strategy to FH diagnosis. We successfully enriched the entire *LDLR* (46.2 kb) and *PCSK9* (26.5 kb) and detected FH mutations in patients with hypercholesterolemia. We also validated a false-positive deletion caused by a long-range PCR.

## Materials and methods

### Patient samples

One family case, four unrelated cases, and one control case are included in this study (Table 1). They are all Japanese. The participants were recruited through the Department of Cardiovascular Medicine of Kyoto University Hospital. The proband in the family case was a 53-year-old mother with an LDL-C level of 227 mg/dL before medication was administered. Statin treatment lowers the LDL-C level to around 170 mg/dL. Her LDL-C level dropped to 68 mg/dL after receiving a PCSK9 inhibitor. Two of her children also exhibited hypercholesterolemia. Her father and elder brother had a history of myocardial infarction. The four independent patients had high initial LDL-C levels (>190 mg/dL) before medication. The Ethics Committee of Kyoto University approved the study. All members provided written informed consent.

**Table 1:** Lipid profile of family cases, unrelated cases and control sample.

	Family Case				Unrelated Cases				Control
	Mother (proband)	Son 1	Son 2	Daughter	Individual # 01	Individual # 02	Individual # 03	Individual # 04	
**Hypercholesterolemia**	Yes	Yes	No	Yes	Yes	Yes	Yes	Yes	No
**Age**	53	19	16	12	65	59	33	41	57
**Gender**	Female	Male	Male	Female	Female	Female	Male	Female	Male
**Total cholesterol (mg/dL)** *	N/A	231	194	289	879	567	327	323	210
**Triglycerides (mg/dL)** *	74	87	50	136	N/A	174	107	127	126
**HDL-cholesterol (mg/dL)** *	43	52	68	41	N/A	59	47	63	58
**LDL-cholesterol (mg/dL)** *	227	158	115	213	763	463	259	232	127
**Medication**	Atorvastatin 40 mg/day + Ezetimibe 10 mg/day	No	No	Pitavastatin 1mg/day	Rosvastatin 15 mg/day + Ezetimib 10 mg/day	Pitavastatin 15 mg/day + Ezetimib 10 mg/day	Rosuvastatin 5 mg/day	N/A	N/A
**LDL-cholesterol (mg/dL)**	170	No	No	116	155	227	136	N/A	N/A
**Adjusted Medication**	Atorvastatin 40 mg/day + Evolocmab 420 mg/month								
**LDL-cholesterol (mg/dL)**	68								

Abbreviations: LDL, low-density lipoprotein; HDL, high-density lipoprotein

*. were pre-treatment levels

### Genomic DNA isolation

About 5 mL of peripheral blood was collected from each person. DNA was extracted according to the manufacturer’s protocol with a PAXgene Blood DNA kit (PreAnalytix, Qiagen, GmbH, Germany). The quality of DNA was checked using a Nanodrop 2000 spectrophotometer (Thermo Fisher Scientific, USA).

### CRISPR-RNAs design

CrRNAs were designed by CRISPick (https://portals.broadinstitute.org/gppx/crispick/public), IDT tool (https://sg.idtdna.com/site/order/designtool/index/CRISPR_CUSTOM), and CHOPCHOP (https://chopchop.cbu.uib.no/). Candidate crRNAs were searched against the human genome (hg38) for common single nucleotide polymorphisms (SNPs) via the UCSC Genome Browser. CrRNAs without common SNPs were further assessed for their on- and off-targeting potential using the IDT CRISPR-Cas9 crRNA checker (https://sg.idtdna.com/site/order/designtool/index/CRISPR_SEQUENCE). CrRNAs with both the on- and off-target performance scores above 50 are considered qualified. A higher score indicates better predicted performance. Finally, 12 crRNAs were decided (see S1 Table for details of the crRNA sequences). We performed triple Cas9-guided cuts on either side of the *LDLR* and *PCSK9*. In the benchmark experiment, we defined ROIs as the regions between the innermost crRNA cutting sites of *LDLR* and *PCSK9* (-623 to *LDLR* +1230 bp, -1231 to *PCSK9* +26 bp, in total, 70,805 bp). It is easier to observe the enrichment results on *target genes* with a broader range. Another reason is that the innermost cleavage site covers the flanking regions on both sides of the target gene. Notably, the upstream regions within several hundred base pairs from genes often contain regulatory elements that could impact gene functions. While this study does not extensively investigate specific regulatory elements, we include these neighboring flanking regions for potential opportunities for refining variant screening in future research. Please refer to S1 Fig for the schematic illustration of crRNAs excision and the ROIs.

Alternatively, we enriched *LDLR* with two sets of crRNAs (overlapping at exons 6–7) and enriched *PCSK9* via four crRNAs out of the previous six. The on-targeting efficiency is similar. For details on this alternative panel, please see S2 Table, S3 and S4 Figs.

### Target cleavage, ONT library preparation, and sequencing

Based on the ONT protocol (ligation sequencing gDNA—Cas9 enrichment version: CAS_9106_v109_revE_16Sep2020, available on ONT Community), the sequencing library was prepared with an ONT sequencing kit (Cat # SQK-CS9109). Five μg gDNA extracted from peripheral blood was first dephosphorylated. The Cas9 ribonucleoprotein complexes synthesized from crRNAs, tracRNA (IDT, Cat #1072532), and Cas9 nuclease (IDT, Cat #1081060) performed double-strand cleavage at the target sites (60 min 37°C incubation). The fresh 5’ ends with exposed phosphate groups at the cutting sites were added to the adenine tails. Sequencing adapters were ligated to the tailed DNA ends (20-min incubation). The excess adapters were removed from the mix using AMPure XP beads (Beckham Coulter, Cat # A63880). The non-target gDNA molecules remained. The prepared library was loaded onto an R9.4 flow cell (ONT, FLO-MIN106) on a MinION Mk1B sequencer. The sequencing was monitored using MinKNOW software (ver. 19.12.5) and run with the "high-accuracy" base-calling mode. Data usually stopped producing between 24 and 36 hours.

Please refer to the S1 File for details on the command lines for the bioinformatic analysis.

### Data processing and alignment

Raw sequencing signals were base-called and converted to FASTQ format reads. We aligned the reads to the GRCh38 human reference genome using Minimap2 [18] (ver. 2.20-r1061) and sorted them using SAMtools [19] (ver. 1.12). Alignments were visualized on the Integrative Genomics Viewer (IGV, ver. 2.11.1). Sequencing data are accessible at DNA Data Bank of Japan (Accession numbers are SAMD00748665 (Benchmark_experiment), SAMD00748666 (Family_mother), SAMD00748667 (Individual_01), SAMD00748668 (Individual_02), SAMD00748669 (Individual_03), and SAMD00748670 (Individual_04).

The released samples are available on the DDBJ BioSample listing page.

https://ddbj.nig.ac.jp/resource/biosample/SAMD00748665

https://ddbj.nig.ac.jp/resource/biosample/SAMD00748666

https://ddbj.nig.ac.jp/resource/biosample/SAMD00748667

https://ddbj.nig.ac.jp/resource/biosample/SAMD00748668

https://ddbj.nig.ac.jp/resource/biosample/SAMD00748669

https://ddbj.nig.ac.jp/resource/biosample/SAMD00748670).

### On/off-target analysis

The unspecific binding of Cas9 complexes and the shearing force during the experiment break gDNA molecules outside of the ROIs, generating sequencing reads elsewhere. We define "off-targets" as "pileups outside of ROIs with a distance > 1000 bp and coverage of > 25x." "Off-target" candidates were picked out using SURVIVOR [20]. The coverage on ROIs, "off-target" sites and the whole genome was calculated using SAMtools to observe the on-target enrichment performance. The on-target ratio was calculated using the number of on-target reads versus the number of total reads.

### Variant calling, annotation and confirmation

We first align the sequencing reads to the human reference genome and use the command line tool Bcftools (ver. 1.13) [19] to identify potential variant sites. Outputs include SNPs, Indels, multi-nucleotide variants and structural variants (SVs). Nanopore sequencing data contains random errors, which could be recognized as potential variants. To filter out true variants, we employ the following two steps: firstly, we annotate all the reported variant sites using the ANNOVAR (Annotation of Genetic Variants, ver. 2019Oct24) [21]. ANNOVAR is a widely used bioinformatics tool that integrates variant annotation information from various databases. We utilize hg38-build databases in our workflow: refGene, Clinvar, avsnp150, and dbnsfp41a. The refGene database annotates variants’ genetic positions, for example, intronic, exonic, splice sites and other relevant genomic features. We have summarized some regulatory elements’ names and genomic coordinates in the *LDLR* upstream gene. These elements are not listed in the refGene annotation but could be used for an expanded search for variants (S3 Table). Those elements’ mutations could potentially alert transcription activity [22, 23]. The Clinvar database contains information on previously reported variants, including their clinical significance. It collects different types of variants, including SNVs, Indels and SVs. The avsnp150 database (also known as dbSNP) offers information on SNPs, including their frequencies and functional annotations. The dbNSFP41a database offers functional impact predictions. After annotation, we filtered out variants labeled as "Pathogenic" and "Likely pathogenic" or predicted as high-risk based on functional predictions.

Secondly, for the selected key variants, we examine the sequencing data of the corresponding positions using the genome visualization tool IGV. In case of a random error during high-speed nanopore sequencing, the variant at that specific site will only be observed in a limited number of reads. It is crucial to verify if the suspicious variant is present in both forward and reverse direction reads. Based on our current experimental expertise, detecting the variant in both directions confirms its existence. If it is supported by reads from only one direction, it is highly likely to be a base-calling error rather than an actual existing variant. Please refer to the schematic diagram in S5 Fig.

Following the above steps, we have filtered out high-risk variants annotated by public databases. We further confirmed the presence of these variants through Sanger sequencing.

We also extracted the continuous long reads overlapping the entire *LDLR* or *PCSK9*. By using these non-amplified native reads, it is possible to assess whether there are any noticeable SVs visually.

### Variant validation

We validated the candidate pathogenic mutations by Sanger sequencing on ABI PRISM 3130xl Genetic Analyzer (Applied Biosystems) using the BigDye Terminator v3.1 Cycle Sequencing Kit (Applied Biosystems, Cat # 4337456). For mutations located in regions where it is challenging to generate a unique amplicon for Sanger sequencing, we performed an in-fusion cloning experiment using a plasmid pcDNA-3.1 vector and In-Fusion^®^ HD Cloning Kit (Takara, Cat #639648), according to the manufacturer’s instructions. We analyzed the variants with Sanger sequencing.

## Results

### *LDLR* and *PCSK9* were successfully enriched

**Fig 1:**
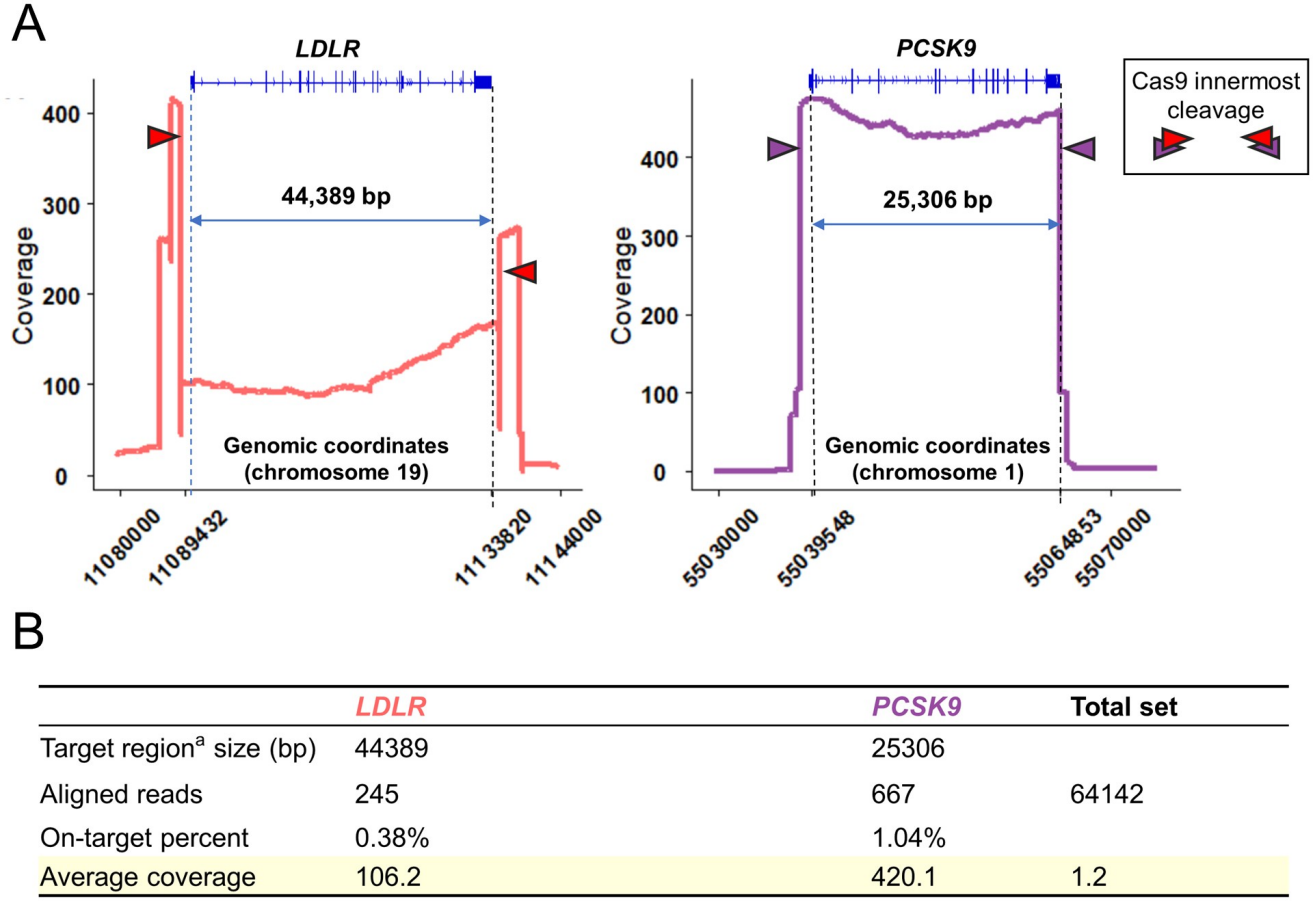
Cas9-mediated enrichment performance on ROIs. (A) The coverage plot at *LDLR/PCSK9* in the benchmark experiment. The ROI areas were referred to each gene’s innermost crRNAs cutting sites. (B) On-ROI percent was calculated using the number of aligned reads on ROIs and the number of reads of the whole genome (64,142).

In the benchmark experiment, we used 5 μg of gDNA for library preparation, followed by 36 hours of sequencing using the crRNAs panel (refer to S2 Table). To assess the enrichment performance of *LDLR/PCSK9*, we defined Regions of Interest (ROIs) as the regions between the innermost crRNA cutting sites of the two genes (-623 to *LDLR* +1230 bp and -1231 to *PCSK9* +26 bp). Among the total 64,142 aligned reads in the dataset, 245 aligned to *LDLR*, and 667 were assigned to *PCSK9* (Fig 1A). The on-ROI ratio was calculated as 0.38% (245/64,142) for *LDLR* and 1.04% (667/64,142) for *PCSK9*. The average coverage for *LDLR* and *PCSK9* was 106× and 420×, respectively, compared to 1.2× for the entire genome (Fig 1B), indicating adequate enrichments.

Additionally, we observed 13 defined "off-target" pileups across the dataset (refer to S4 Table). These off-target coverages ranged from 25× to 41×, significantly lower than the ROI coverages, indicating that the on-ROI enrichment was adequate.

We detected annotated pathogenic (or likely pathogenic) mutations in other samples, which will be reported in the following results. We also calculated their *LDLR/PCSK9* coverage (see S5 Table and S2 Fig).

### Continuous reads rule out a false-positive 670 bp deletion due to long-range PCR error

**Fig 2:**
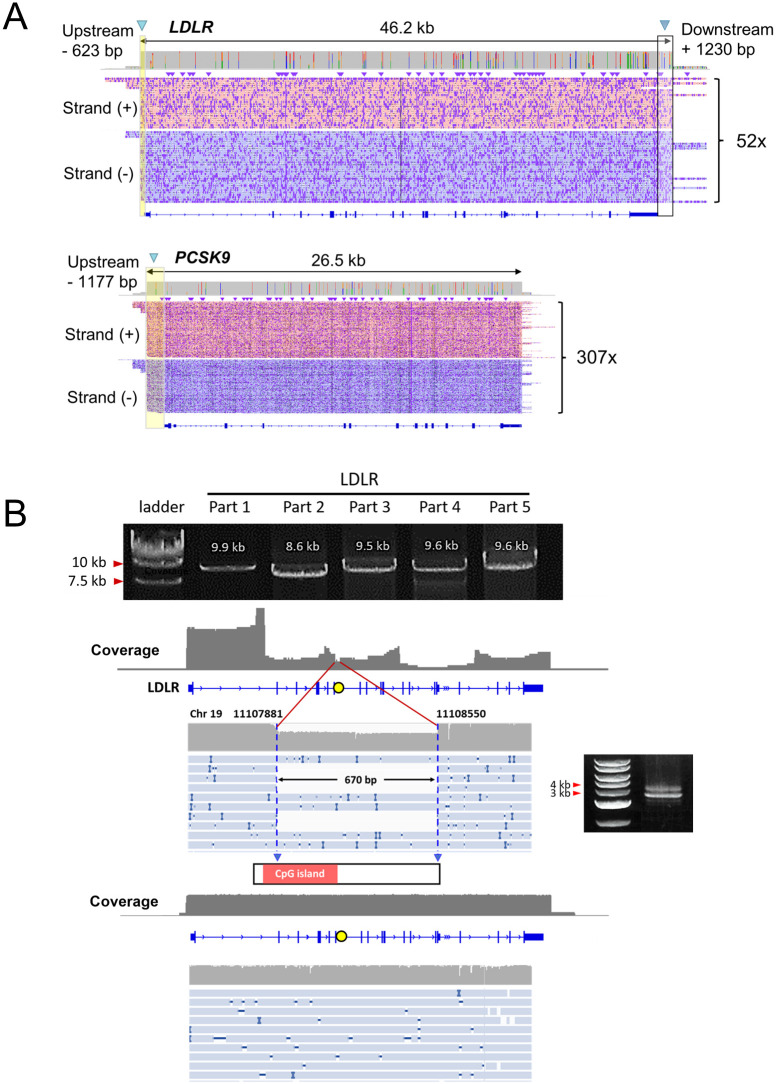
Continuous long reads generated by this strategy rule out a false-positive 670 bp deletion due to PCR error. (A) Coverage on ROIs of full-length reads spanning the whole *LDLR* and *PCSK9*. (B) The continuous long read sequences proved that the 670 bp deletion within the *LDLR* gene was a false positive result caused by PCR amplification error. We used the 2.5 kb DNA ladder (Takara, Cat # 3413A) as a DNA molecular size marker in agarose gel electrophoresis. The band of biased amplicons (~8.9 kb) of part three was challenging to distinguish from the band of complete amplicons (~9.5 kb). It was only noticeable by shorter amplicons and a smaller-scale DNA ladder. With the continuous long reads generated by Cas9-guided nanopore sequencing, it was easy to see no deletion in the relevant region.

Amongst the on-ROI reads obtained in the benchmark experiment, continuous reads spanning *LDLR* and *PCSK9* were observed in 21% (52/245) and 48% (307/667), respectively (Fig 2A). These uninterrupted reads are highly useful for detecting SVs.

Before establishing this Cas9-guided sequencing workflow, we attempted to detect genetic variants in *LDLR* through long-range PCR. We divided *LDLR* into five segments and amplified each part using long-range PCR. Subsequently, we sequenced the amplicons of these five segments to identify mutations (PCR primers and reaction conditions can be found in the S6 Table).

However, even with high-fidelity PCR enzymes claimed to have high amplification efficiency, the high GC% or low complexity regions within *LDLR* still cause amplification troubles. Previous PCR experiments involving other samples reported a 670 bp deletion (chr19:11107881–11108550). This variation appeared in multiple unrelated samples, making it most likely a false positive finding. In this study, we performed the same PCR amplification on the control sample used in the benchmark experiment. It also exhibited this 670 bp deletion. The deletion could not be distinguished from the 9.5 kb PCR amplicon but was noticeable through agarose gel electrophoresis in a shorter amplicon. The problematic site is located in a 295 bp CpG island in intron six of *LDLR*. Those original continuous reads bypass the influence of difficult regions within the gene on PCR amplification, clearly indicating that the 670 bp deletion does not exist at that reported site (Fig 2B).

### A heritable FH mutation was detected in a family case

**Fig 3:**
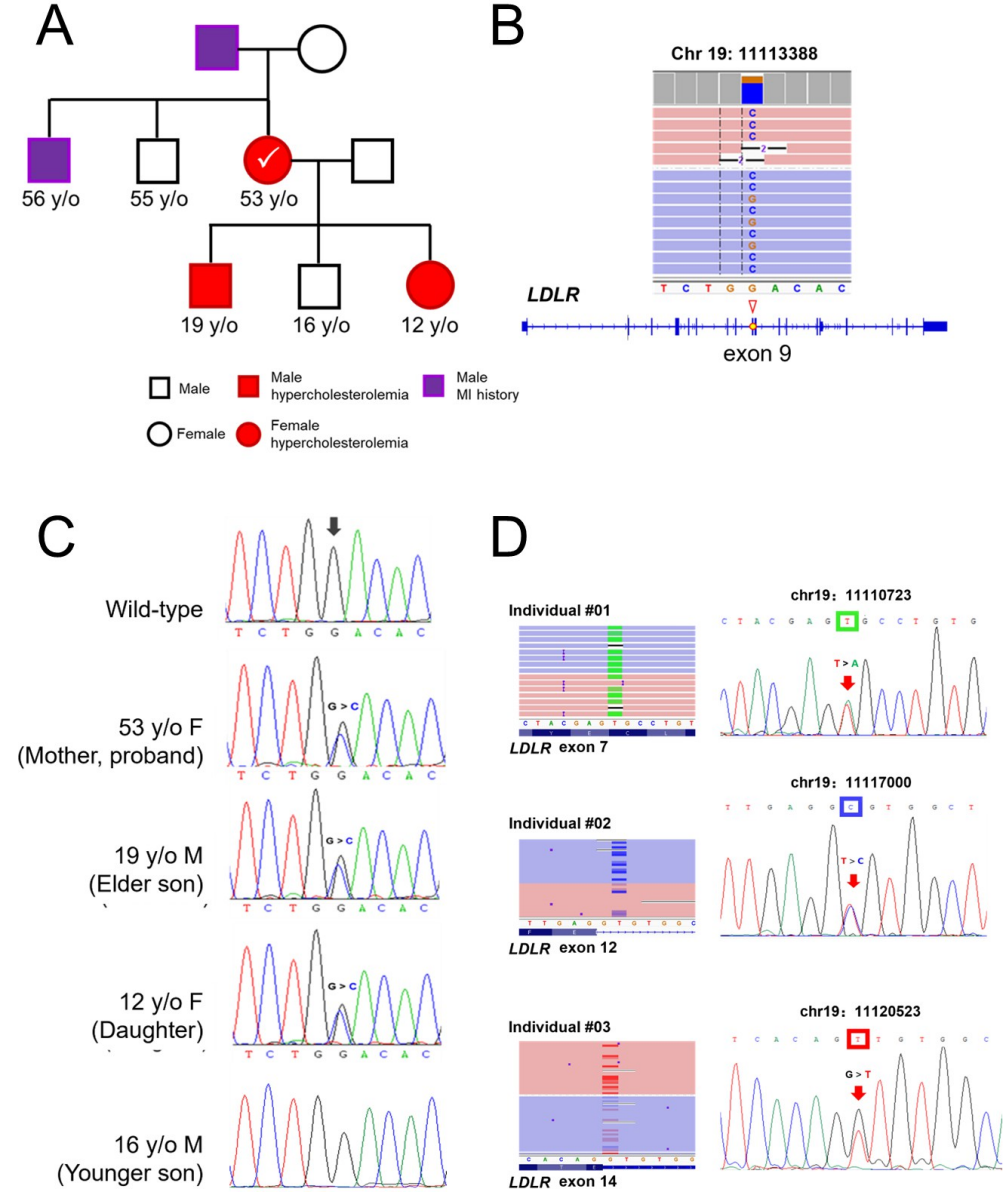
FH-causative mutations were detected in the *LDLR*. (A) Pedigree of a family with hereditary hypercholesterolemia. The proband case (mother) and two of her three children (19-year-old son, 12-year-old daughter) exhibited hypercholesterolemia. The proband’s father and elder brother had a myocardial infarction history. (B) The mutation c.1297G>C: p. (Asp433His) is located in exon 9 of the *LDLR* gene. Visualization tool IGV showed that both strands supported the mutation. (C) Sanger sequencing confirmed this mutation happened in the proband patient and the two children with hypercholesterolemia. The other 16-year-old son, without hypercholesterolemia, was free of this mutation. (D) Via the same variant filtering strategy, three heterozygous SNVs were detected in other unrelated cases. Those variants were supported by both strands and were confirmed by Sanger sequencing.

We performed sequencing and variant screening of *LDLR* and *PCSK9* in a 53-year-old female patient with hypercholesterolemia (Fig 3A). A reported mutation [24] was identified. It was c.1297G>C: p. (Asp433His) in *LDLR* exon nine (Fig 3B). We screened her three children (Fig 3A) using Sanger Sequencing to validate the inheritance of that mutation. The proband’s 19-year-old son and 12-year-old daughter were found with the same mutation. The 16-year-old son, without hypercholesterolemia, was free of this mutation. The mutation was confirmed via Sanger sequencing (Fig 3C).

### Pathogenic SNVs were found in another three cases

FH-causative SNVs were found in three unrelated patients with hypercholesterolemia (Table 1). All the mutations were previously reported in other studies and classified as pathogenic or likely pathogenic (Table 2). One was a missense mutation located in *LDLR* exon 7. Mutations were found in introns 12 and 14 of the *LDLR* (Fig 3D). Both of them were located at splice donor sites.

**Table 2:** FH-causative SNVs were detected in three unrelated cases with hypercholesterolemia.

Individual	dbSNP ID	Location (chr19)	Gene	Region of function	Nucleotide change	Consequence	Clinical significance
#01	rs879254753	11110723	*LDLR*	Exon 7	c.1012T>A	Missense (Cys338Ser)	Pathogenic/likely pathogenic
#02	rs778408161	11117000	*LDLR*	Intron 12	c.1845+2T>C	Splice donor variant	Pathogenic
#03	rs145787161	11120523	*LDLR*	Intron 14	c.2140+1G>T	Splice donor variant	Pathogenic/likely pathogenic

All mutations were previously reported by other studies.

### An 11,029 bp novel deletion was identified

**Fig 4:**
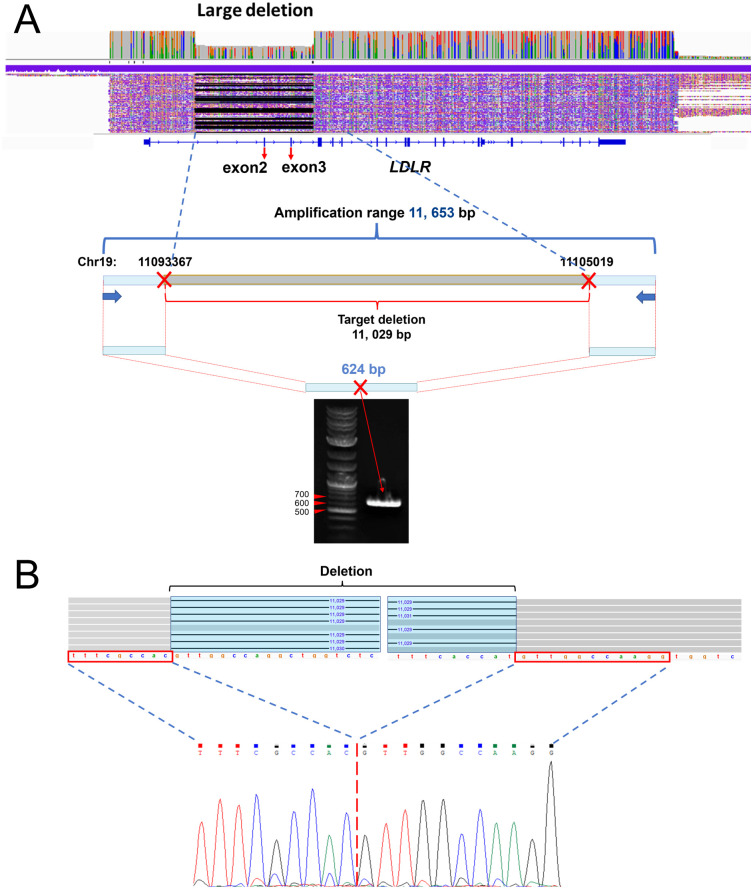
An 11,029 bp deletion in *LDLR* was identified. (A) Continuous long reads clearly show the deletion on IGV. By amplifying the flanking region of the deletion, a 624 bp short amplicon containing the breaking point was confirmed by gel electrophoresis. (B) The deletion was further confirmed on the sequence level.

A heterozygous novel deletion was found in the *LDLR* of patient #04. The continuous long reads showed that the deletion was located in chromosome 19: 11093597–11104625, skipping exons 2–3 (Fig 4A). We confirmed the deletion through an in-fusion cloning experiment (Fig 4B).

## Discussion

Identification of causative variants is crucial for precision medicine in FH patients and their families. This study integrated the CRISPR-Cas9 system with nanopore sequencing technologies to address clinical demands. We developed novel crRNA panels (S1 and S2 Tables) and successfully achieved targeted enrichment of *LDLR* and *PCSK9* without amplification (Fig 1). Through this approach, we identified multiple mutations of various types and sizes. The workflow can be easily implemented in a small laboratory using a portable sequencer and laptop computer, allowing completion by one person within 1.5 to 2 days (including 2 hours for DNA extraction, 3 hours for library preparation, and 24 to 36 hours for sequencing). Please refer to the S7 Table for details.

In the benchmark experiment, the on-ROI read ratios of *LDLR* and *PCSK9 were* 0.38% and 1.04%, which might appear low. The on-ROI ratios were calculated with the entire genomic DNA background, while the background DNA molecules were not removed from the targets during the purification step. Commonly, the coverage required for variant calling from Nanopore sequencing is several tens-fold [39]. The average coverages at *LDLR* and *PCSK9* were 106x and 420x (Fig 2B). As for the continuous reads, the coverages were 52x and 307x, respectively (Fig 2A). Thus, we considered the enrichment performance to be generally satisfactory. As for *PCSK9*, the enrichment performance even exceeded our expectations. We need to sample down reads to save unnecessary computational power during variant calling.

The coverage of *LDLR* was lower than *PCSK9* (Fig 1, S2 Fig). This observation is consistent with the benchmark experiment results. There are several possible reasons for this. ONT recommends targeting lengths not exceeding 20 kb due to the propensity of long DNA molecules to break. Otherwise, it will result in a decreasing read count towards the center of the target region. *LDLR* length surpasses the recommendation by more than two-fold. Considering the inherent advantages of long-read sequencing in detecting SVs, we were challenged to cut the *LDLR* and *PCSK9* only at both sides for uninterrupted gene enrichment. As expected, long targets have decreased coverage toward the middle of the ROIs (Fig 1A, S1, S2 Figs). Another possible reason is that the repetitive regions upstream of *LDLR* restrict crRNA selection. After the benchmark experiment, we conducted sequencing for Family-mother and Individual 1 and discovered that pre-mixing the 12 crRNAs could improve experimental efficiency. Subsequently, we purchased the same crRNAs and divided them into separate portions to avoid repeated freeze-thaw cycles. This approach effectively enhanced enrichment efficiency for subsequent samples. Similar improvements can be observed in *PCSK9* data as well. More notably, we found that some Cas9 complexes for *LDLR* targeted more reads of opposite directions than expected, causing a sharp decrease in coverage between the innermost cutting sites. The Cas9 complexes may have caused unintended cleavage at nearby off-target locations, resulting in the retention of reads from opposite directions. Structural complexities in the *LDLR*, such as hairpin loops or secondary structures, can also hinder efficient cleavage by the Cas9-complexes. This is a reminder that although all candidate crRNAs for *LDLR* (with one exception) scored > 50 for both on- and off-target performance, the actual implementation can still vary. CrRNAs for *PCSK9* generally scored higher for on-target predictions and had better enrichment performance, indicating that a high on-target score is more important in enrichment success.

Our study defined "off-targets" as "pileups occurring outside of ROIs with a distance greater than 1,000 bp and coverage exceeding 25x". We identified 13 such "off-targets" in the benchmark experiment. The purpose of presenting their locations and coverage alongside the enrichment results of on-ROI samples was to facilitate comparison. However, since our research does not involve gene editing, off-target reads outside the ROIs are not particularly problematic for mutation detection. Analysis tools can easily exclude them.

We successfully ruled out a tricky PCR error (Fig 2B). Previously, we sequenced *LDLR* by long-range PCR amplicons with five primer pairs. In long-range PCR, the Takara PrimeSTAR GXL DNA Polymerase was considered highly accurate in high GC% regions [11]. However, designing long-range PCR primers and ensuring amplification accuracy for *LDLR* remains a formidable challenge. The 670 bp deletion reported in our study serves as such an example. The deletion occurred in many samples under the same PCR conditions. Thus, it is considered highly possible to be a false positive.

The control sample used in the benchmark experiment also showed this PCR error under the same conditions mentioned in the S6 Table. The continuous long reads generated by our strategy were directly sequenced without any amplification. It is quickly determined that the deletion does not exist.

Regarding mutation identification, we successfully identified a hereditary pathogenic SNV (c.1297G>C) within a family case. This mutation occurs in the epidermal growth factor (EGF) precursor homology domain of *LDLR* and induces an alteration in amino acids [24]. The mutation leads to impaired maturation and accelerated degradation of the mature LDLR protein. The LDLR EGF-precursor homology domain, spanning exons 7–14 and consisting of 411 amino acids, plays a crucial role in lipoprotein release and stabilization of the LDL receptor [25–28]. Despite inadequate response to conventional statin therapy, the proband patient exhibited sensitivity towards PCSK9 inhibitors which partially mitigated PCSK9-induced degradation of LDLR.

We applied the workflow to more cases and found FH-causative SNVs in another three unrelated hypercholesterolemic patients. Previous studies show that intronic mutations in the *LDLR* could also result in the FH phenotype. Most of these mutations are located within 20 bp of exon-intron boundaries, and extensive screening of deeper introns is not usually performed [29]. However, intron mutations that locate hundreds or even thousands of bps away from exon-intron boundaries have also been reported to cause disease phenotypes [8, 30, 31], which indicates the need to expand the sequencing for patients whose genetic cause of FH remains unknown. The length of *LDLR* is approximately 46.2 kb, which exceeds the amplification capacity of conventional long-range PCR polymerases (<40 kb) [11]. However, using our method, we were able to enrich reads covering the entire *LDLR* gene, including intronic regions that are far from the exons. This provides a convenient means for screening for the aforementioned intronic variations. Also, regardless of the complexity of the genomic region, our variant filtering strategy enables filtering for different variants, including SNPs, SNVs, Indels, SVs, etc. Of course, our research still has limitations.

Nanopore long reads are capable to identify mutations of various sizes [32–35], as shown in our results. However, due to the limited sample size, we reported only SNVs, one large deletion this time. For the annotated putative pathogenic genes, the recall rate was 100%. Unfortunately, we are currently unable to provide precise estimates for the method’s large-scale application. To address this limitation, we plan to expand our sample size and gather further evidence through ongoing research.

In this study, we aimed to establish the Cas9-guided amplification-free workflow and accumulating evidence from real-world cases, so only included *LDLR* and *PCSK9* this time. Other relevant genes including *APOB* and *APOE* also contribute to the FH phenotype. By following the similar crRNA designing principle, more genes can be added to the enrichment panel and targeted simultaneously. We plan to expand the current gene panel in future work.

Currently, our primary focus is on identifying known pathogenic or likely pathogenic variations that are already documented in public databases. For unreported variations, we use genetic position annotation as a preliminary screening method, considering factors such as their presence in or proximity to protein-coding regions or regulatory regions. Experimental validation is then required to confirm the pathogenicity of these variations. In other words, our current analysis workflow is particularly efficient in confirming reported mutations and visually confirming SVs rather than identifying de novo small-scale variations.

Our method has low initial costs and minimal equipment and labor requirements, making it highly suitable for small-scale laboratories. However, the cost per sample is still higher compared to PCR or NGS. The current workflow uses one ONT flow cell per sample, which costs approximately $600, including consumables. The average cost of materials for a short-read whole-genome sequence (WGS) is approximately $1,000. ONT has already released 24 extended barcodes that can sequence multiple samples in a single experiment, but they mainly support amplification-based sequencings. We are currently working on barcoding sequencing using different patterns of DNA sequences as markers and attempting to separate the data of different samples based on this during the analysis stage. We expect to facilitate sequencing multiple samples in a single experiment, thereby reducing overall costs. Based on our experience so far, our approach is especially effective in rapidly screening suspected variations in *LDLR/PCSK9* among patients displaying obvious FH phenotypes or with a family history of the condition rather than for large-scale population screening.

The long reads obtained from non-amplification DNA molecules hold great potential for uncovering new diagnostic clues for FH. For example, a study shows that FH-mutation-negative patients are characterized by the accumulation of differential DNA methylation throughout the genome [36]. Sequencing data generated through amplification-free nanopore sequencing can keep methylation information [37, 38]. Continuous reads also allow analyzing deep intronic regions, microRNA binding sites in the 3’-UTR, or exploring SNP distribution patterns.

Our current variant filtering strategy allows us to filter for variants already collected in the public databases. We plan to expand the sample size and incorporate more databases into the annotation step with regular updates. In addition, there has been a proliferation of analysis tools designed to screen for variations in long nanopore sequencing data. These include tools for calling small variants (e.g., PEPPER-Margin-Deep Variant, Medaka, Clair3) and identifying SVs (e.g., Sniffles, cuteSV). ONT also continuously updates its hardware to reduce random errors and improve sequencing accuracy in low-complexity regions. For example, the latest ONT R10.4 series flowcell has been reported to have significantly better accuracy in homopolymers [39].

In conclusion, we established a new workflow that combines the CRISPR-Cas9 cleavage system and nanopore sequencing for FH genetic testing. We sincerely hope this study serves as a starting point for future research and contributes to identifying more FH cases.

## Supporting information

S1 Table CrRNA sequences used for *LDLR/PCSK9* targeting cleavage. (PDF)

S2 Table Alternative crRNA panel used for *LDLR/PCSK9* targeting cleavage. (PDF)

S3 Table The upstream regulatory elements of *LDLR* involved in our study. The reported regulatory elements within 280 bp from *LDLR* transcription starting site.
(PDF)

S4 Table Off-target pileups from the whole benchmark experiment data set. (PDF)

S5 Table Average coverage of LDLR/PCSK9 for other samples. (PDF)

S6 Table The PCR primers and reaction conditions to amplify *LDLR*. (PDF)

S7 Table Estimated time required for a single experiment. (PDF)

S1 Fig Excision pattern for *LDLR/PCSK9* (Schematic diagram). (PDF)

S2 Fig Average coverage of *LDLR/PCSK9* for main samples reported in this study. The figure includes the proband case in the family analysis (Family-mother), three unrelated cases with detected SNVs (Individual 1, 2, 3), and the case with a large deletion (Individual 4). The zoomed-in image in the middle represents the *LDLR* coverage for two samples: Family-mother and Individual 1.
(PDF)

S3 Fig Alternative crRNA targeting strategy for *LDLR/PCSK9*. The alternative target strategy: enriching *LDLR* with two sets of crRNAs (ROIs overlap at exons 6 and 7) and enriched *PCSK9* using four crRNAs out of the previous six. The color blocks refer to the regions between the innermost cutting sites that are targeted.
(PDF)

S4 Fig The enrichment patterns of *LDLR/PCSK9* using the alternative crRNA panel. (PDF)

S5 Fig Evidence of variant existence confirmed by forward and reverse read. The random errors due to the high-speed nanopore sequencing process often appear in a few reads or only in reads from one direction. The presence of variants supported by reads from both directions strongly indicates their existence.
(PDF)

S6 Fig Three gel images for Figs 2B and 4A. (PDF)

S1 File Bioinformatic analysis command lines. (PDF)
