# Supplementary material for: CRISPR-Cas9-guided amplification-free genomic diagnosis for familial hypercholesterolemia using nanopore sequencing
Source: PLoS One. 2024 Mar 20;19(3):e0297231. doi: 10.1371/journal.pone.0297231 (PMC10954175; doi:10.1371/journal.pone.0297231)
Supplement: S2 Table — (PDF) [file pone.0297231.s002.pdf]

S2 Table. Alternative crRNA panel used for *LDLR*/*PCSK9* targeting cleavage.

Target gene	Sequence 5'-3'	Cleavage site	Sequence (pam site)	Gene-flanking distance (bp)	Prediction score (on/off-target) *
LDLR	ldlr-f3733	ch19:(+) 11085716	ATGTAAGCCAAGTGCCTTCC(GGG)	-3733	60/56
	ldlr-f1952	ch19:(+) 11087480	GTCGCAAATGGCATAAGGAA(TGG)	-1952	56/51
	ldlr_f623	ch19:(+) 11088809	AATATTTACGTCCAGACTCC(AGG)	-623	68/68
	ldlr_r1230	ch19:(+)11135050	TGTAGTGTCCCACAAGCCTA(CGG)	1230	74/62
	ldlr-r4203	ch19:(-) 11138023	CGCTTATCCTGTTTGCCACT(TGG)	4203	50/69
	ldlr-r4624	ch19:(-) 11138444	GCCAGCAGCGCATTTTACCA(GGG)	4624	30/78 **
PCSK9	pcks9-f2229	chr1:(+) 55037319	ACGATTCAACCCACTACCGT(GGG)	-2229	91/75
	pcks9-f1654	chr1:(+) 55037894	GTTGACCCAGAAAGCACTTG(TGG)	-1654	70/55
	pcks9-f1231	chr1:(+) 55038317	TGGGTGCACGGTAACGACCC(GGG)	-1231	62/92
	pcks9-r26	chr1:(-) 55064879	TATAAGCAAGGCATCACCCT(AGG)	26	83/53
	pcks9-r712	chr1:(-) 55065565	CTGGAGGTCCCACATTGAAT(GGG)	712	90/62
	pcks9-r1382	chr1:(-) 55066235	ACACTAATAAAGGGGACTGT(GGG)	1382	80/53

*. On/off-target performance was scored by IDT CRISPR-Cas9 gRNA checker. The expected score is > 50. A higher score indicates better cutting efficacy or lower off-target risk.

**. Those four sequences are located within *LDLR* gene.
