# Supplementary material for: CRISPR-Cas9-guided amplification-free genomic diagnosis for familial hypercholesterolemia using nanopore sequencing
Source: PLoS One. 2024 Mar 20;19(3):e0297231. doi: 10.1371/journal.pone.0297231 (PMC10954175; doi:10.1371/journal.pone.0297231)
Supplement: S3 Table — The reported regulatory elements within 280 bp from LDLR transcription starting site. (PDF) [file pone.0297231.s003.pdf]

S3 Table. The upstream regulatory elements of *LDLR* involved in our study

Element	Sequence	Chromosome	Start	Stop	Distance (bp) to LDLR (chr19:11089432)	Distance (bp) to transcription starting site (chr19: 11089549)
FP2	GCCTGCCCTGGCG	chr19	11089269	11089281	-163	-280
FP1	GAGCTTCACGGGTTAAAAG	chr19	11089311	11089330	-121	-238
SREBP binding site	TCGGCCGTTCGAAACT	chr19	11089342	11089357	-90	-207
SP1 binding site	CAAACTCCTCCCCCTGC	chr19	11089403	11089419	-29	-146
TATA BoX	TTGAAATGCTGTAAAT	chr19	11089433	11089448	+1	-116
SIRE	GCTGTAAATGACGTGG	chr19	11089440	11089455	8	-109

These are reported regulatory elements within 280 bp from *LDLR* transcription starting site.

Abbreviations: FP, footprint; SREBP1, sterol-regulating element-binding protein; SP, specificity protein; SIRE, sterol regulatory element.
