# Supplementary material for: CRISPR-Cas9-guided amplification-free genomic diagnosis for familial hypercholesterolemia using nanopore sequencing
Source: PLoS One. 2024 Mar 20;19(3):e0297231. doi: 10.1371/journal.pone.0297231 (PMC10954175; doi:10.1371/journal.pone.0297231)
Supplement: S4 Table — (PDF) [file pone.0297231.s004.pdf]

S4 Table. Off-target pileups from the whole benchmark experiment data set.

Chromosome	Start	Stop	Average coverage	
chr1	55039548	55064853	420	***PCSK9***
chr1	143213231	143220195	28	
chr3	196517105	196546968	36	
chr5	19078608	19105589	41	
chr9	4104483	4122151	33	
chr9	17941170	17967605	37	
chr9	99758174	99786021	37	
chr11	31754493	31770366	31	
chr11	67410083	67441577	38	
chr16	46384821	46396348	31	
chr17	64660383	64669679	27	
chr17	68094463	68103414	25	
chr19	11089432	11133820	106	***LDLR***
chr22	20954986	20967725	32	
chrM	3489	10050	25	

Colored items are on-target sites.

Off-target is defined as “pileups outside ROIs with a distance > 1000 bp and coverage > 25x.”
