# Supplementary material for: CRISPR-Cas9-guided amplification-free genomic diagnosis for familial hypercholesterolemia using nanopore sequencing
Source: PLoS One. 2024 Mar 20;19(3):e0297231. doi: 10.1371/journal.pone.0297231 (PMC10954175; doi:10.1371/journal.pone.0297231)
Supplement: S5 Table — (PDF) [file pone.0297231.s005.pdf]

S5 Table. Average coverage of *LDLR/PCSK9* for other samples

	LDLR (Chr19: 11089463-11133820)	PCSK9 (Chr1: 55039548-55064853)
Family-mother	13	92
Individual 1	13	74
Individual 2	277	550
Individual 3	95	209
Individual 4	85	234

We have calculated the average coverage of *LDLR/PCSK9* for the reported samples in this study: Family-mother: the proband case in the family analysis, Individual 1, 2, 3: three unrelated cases with detected SNVs , Individual 4: the case with the 11,029 bp deletion.
