# Supplementary material for: CRISPR-Cas9-guided amplification-free genomic diagnosis for familial hypercholesterolemia using nanopore sequencing
Source: PLoS One. 2024 Mar 20;19(3):e0297231. doi: 10.1371/journal.pone.0297231 (PMC10954175; doi:10.1371/journal.pone.0297231)
Supplement: S6 Table — (PDF) [file pone.0297231.s006.pdf]

S6 Table. The PCR primers and reaction conditions to amplify *LDLR.*

Primer ID	Primer sequence (5'->3')	Genomic location (GRCh38)	Tm (℃)	Amplicon length (bp)
LDLR part 1	CGAGAAATTTCAGGAGGATCTTTCA	Chr19: 11089175-11099058	59.1	9884
	CACCTTGGAAAAGAGATCAAAGTCT		59.2	
LDLR part 2	TAAGTGCATAGTGCATGCTGAGATAAG	Chr19: 11097944-11106540	61.4	8597
	GATTTTCTCAAGAAGAGACAACCAGAG		60.1	
LDLR part 3	CTTGAGAAAATCAACACACTCTGTC	Chr19: 11106529-11116031	58.6	9503
	TGCAACGAATTCTGGAAGAAACAG		60.3	
LDLR part 4	CACATTTGGAGTTTGGGGTTCC	Chr19: 11115955-11125570	60.0	9616
	GGTCCACTGGCTCCAATAAAACTT		61.3	
LDLR part 5	GCCTGGCCACTTACTTTTGTAAATA	Chr19: 11125522-11135083	59.8	9562
	CATTTCTTATATCCCCTGTAGTGTCC		58.8	

PCR condition:

Enzyme: PrimeSTAR GXL (Takara, Cat #R050Q), Input DNA: 500 ng/50 μl

98℃ 10s ⎫ x 40 cycles

68℃ 3m20s ⎭
