# Supplementary material for: CRISPR-Cas9-guided amplification-free genomic diagnosis for familial hypercholesterolemia using nanopore sequencing
Source: PLoS One. 2024 Mar 20;19(3):e0297231. doi: 10.1371/journal.pone.0297231 (PMC10954175; doi:10.1371/journal.pone.0297231)
Supplement: S7 Table — (PDF) [file pone.0297231.s007.pdf]

S7 Table. Estimated time required for a single experiment.

Experiment step	Time cost (h)
Genomic DNA extraction	2
Sequencing library preparation *	3
Sequencing	24–36
Data analysis	0.5
Mutation validation **	6
**Total**	1.5–2 days

*. Based on ONT protocol (ligation sequencing gDNA - Cas9 enrichment version: CAS_9106_v109_revE_16Sep2020).

We routinely incubate the sample for 60 minutes in the Cas9-guided cleavage step (protocol suggests 15~60min) and extend the adapter ligation step to 20 min (protocol suggests 10 min).

**. Sanger sequencing
