# Supplementary material for: CRISPR-Cas9-guided amplification-free genomic diagnosis for familial hypercholesterolemia using nanopore sequencing
Source: PLoS One. 2024 Mar 20;19(3):e0297231. doi: 10.1371/journal.pone.0297231 (PMC10954175; doi:10.1371/journal.pone.0297231)
Supplement: S1 Fig — (PDF) [file pone.0297231.s008.pdf]

# S1 Fig. Excision pattern for *LDLR/PCSK9* (Schematic Diagram).

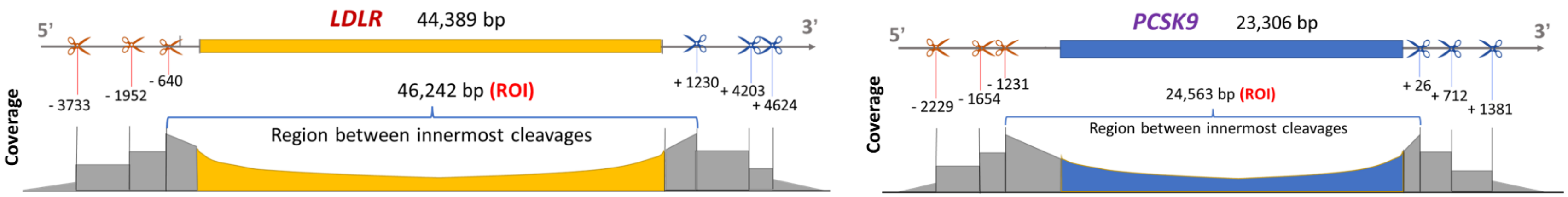


Cleavage sites

| Orientation | LDLR upstream (bp) | LDLR downstream (bp) |
|---|---|---|
| sense | -3733 | |
| sense | -1952 | |
| sense | -623 | |
| antisense | | + 1230 |
| antisense | | + 4203 |
| antisense | | + 4624 |

innermost cleavages

Cleavage sites

| Orientation | PCSK9 upstream (bp) | PCSK9 downstream (bp) |
|---|---|---|
| sense | -2229 | |
| sense | -1654 | |
| sense | -1231 | |
| antisense | | + 26 |
| antisense | | + 712 |
| antisense | | + 1382 |

innermost cleavages

We designed three pairs of crRNA probes for each gene, resulting in three excisions on each side of the gene. As depicted in the figure, it can be observed that the coverage gradually decreases towards the middle of the ROI in real-world scenarios. This decline in coverage is influenced by factors such as the efficiency of cleavage and the length of the target gene and input DNA.

Regions of Interest (ROIs) were defined as the regions between the innermost crRNA cutting sites of the two genes, which cover the flanking regions on both sides of the target gene.:

*LDLR:* upstream 623 bp + gene 44,389 bp + downstream 1230 bp = 46,242 bp

*PCSK9:* upstream 1231 bp + gene 23,306 bp + downstream 26 bp = 24,563 bp.

The total ROIs were 70,805 bp.
