# Supplementary material for: CRISPR-Cas9-guided amplification-free genomic diagnosis for familial hypercholesterolemia using nanopore sequencing
Source: PLoS One. 2024 Mar 20;19(3):e0297231. doi: 10.1371/journal.pone.0297231 (PMC10954175; doi:10.1371/journal.pone.0297231)
Supplement: S2 Fig — The figure includes the proband case in the family analysis (Family-mother), three unrelated cases with detected SNVs (Individual 1, 2, 3), and the case with a large deletion (Individual 4). The zoomed-in image in the middle represents the LDLR coverage for two samples: Family-mother and Individual 1. (PDF) [file pone.0297231.s009.pdf]

S2 Fig. Average coverage of *LDLR*/*PCSK9* for main samples reported in this study.

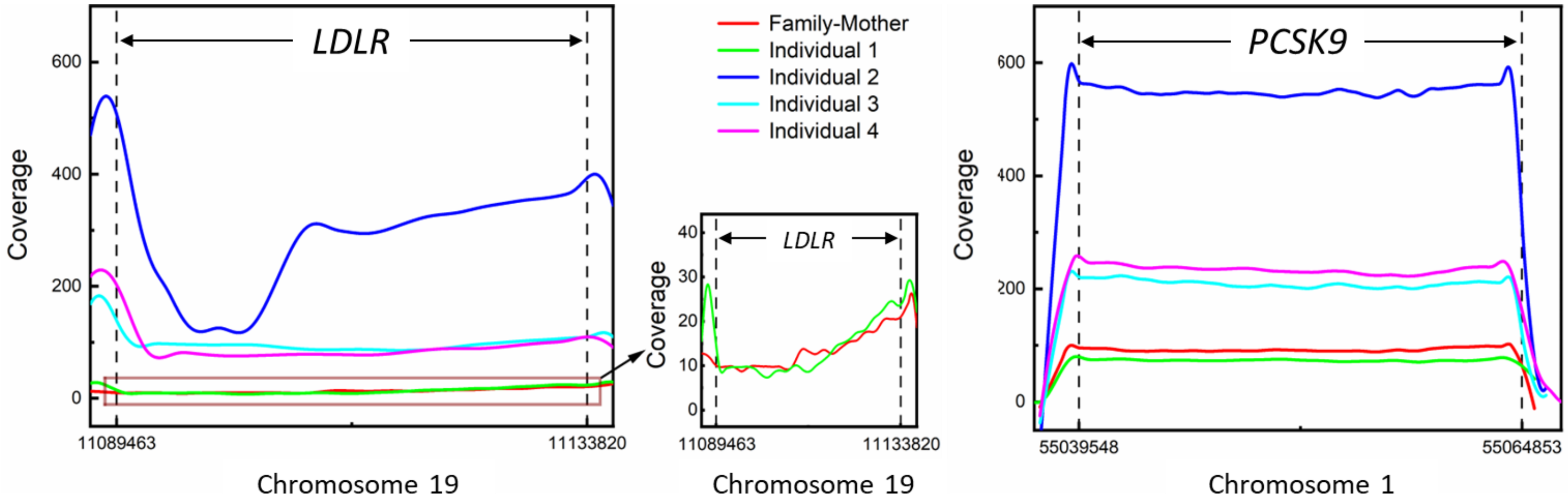

The figure includes the proband case in the family analysis (Family-mother), three unrelated cases with detected SNVs (Individual 1, 2, 3), and the case with a large deletion (Individual 4).

The zoomed-in image in the middle represents the *LDLR* coverage for two samples: Family-mother and Individual 1.
