# Supplementary material for: CRISPR-Cas9-guided amplification-free genomic diagnosis for familial hypercholesterolemia using nanopore sequencing
Source: PLoS One. 2024 Mar 20;19(3):e0297231. doi: 10.1371/journal.pone.0297231 (PMC10954175; doi:10.1371/journal.pone.0297231)
Supplement: S3 Fig — The alternative target strategy: enriching LDLR with two sets of crRNAs (ROIs overlap at exons 6 and 7) and enriched PCSK9 using four crRNAs out of the previous six. The color blocks refer to the regions between the innermost cutting sites that are targeted. (PDF) [file pone.0297231.s010.pdf]

S3 Fig. Alternative crRNA targeting strategy for *LDLR*/*PCSK9.*

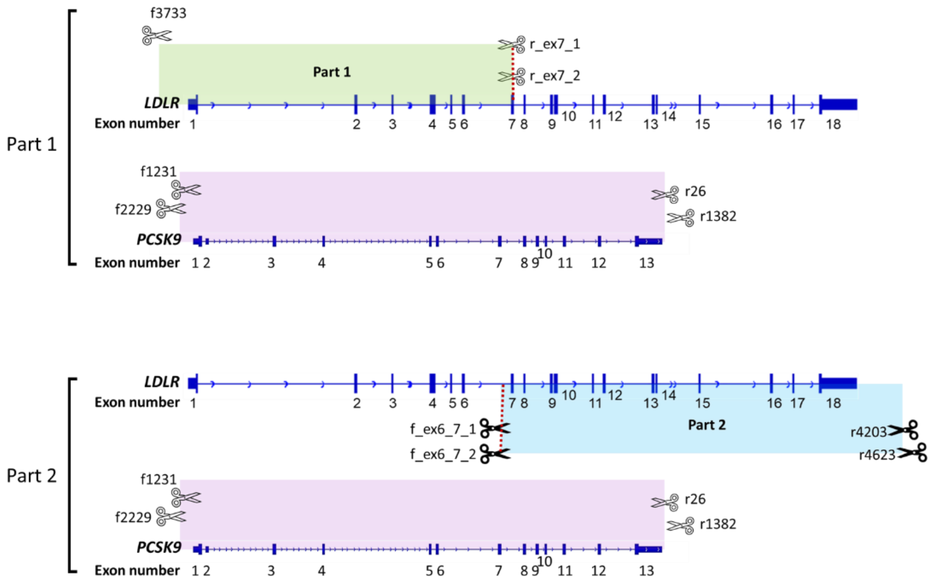

The alternative target strategy: enriching *LDLR* with two sets of crRNAs (ROIs overlap at exons 6 and 7) and enriched *PCSK9* using four crRNAs out of the previous six. The color blocks refer to the regions between the innermost cutting sites that are targeted.
