# Supplementary material for: CRISPR-Cas9-guided amplification-free genomic diagnosis for familial hypercholesterolemia using nanopore sequencing
Source: PLoS One. 2024 Mar 20;19(3):e0297231. doi: 10.1371/journal.pone.0297231 (PMC10954175; doi:10.1371/journal.pone.0297231)
Supplement: S4 Fig — (PDF) [file pone.0297231.s011.pdf]

S4 Fig. The enrichment patterns of *LDLR/PCSK9* using the alternative crRNA panel.

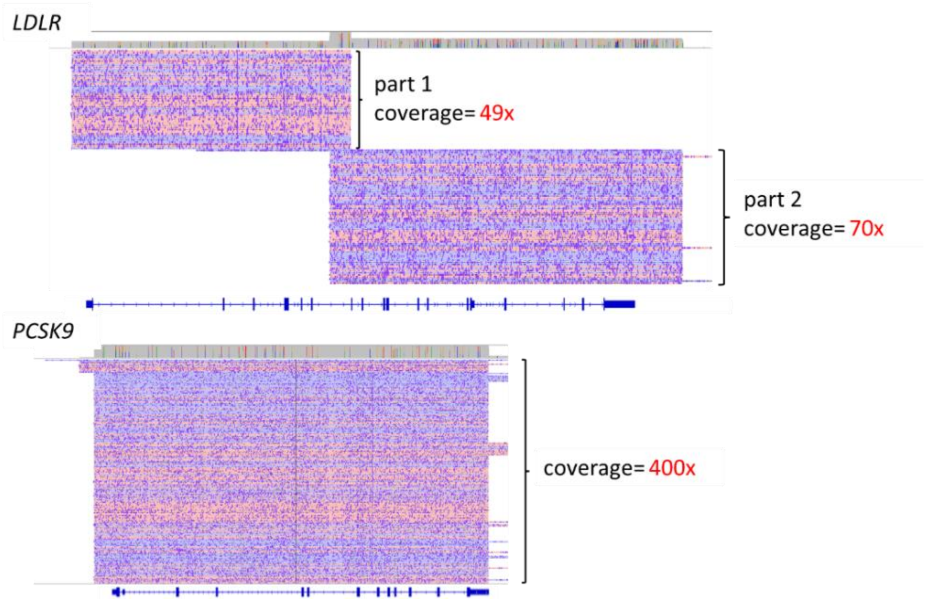

We enriched the LDLR/PCSK9 of the control sample using the alternative crRNA panel. The coverage was calculated using continuous reads.

The result was visualized on Integrative Genomics Viewer.
