# Supplementary material for: CRISPR-Cas9-guided amplification-free genomic diagnosis for familial hypercholesterolemia using nanopore sequencing
Source: PLoS One. 2024 Mar 20;19(3):e0297231. doi: 10.1371/journal.pone.0297231 (PMC10954175; doi:10.1371/journal.pone.0297231)
Supplement: S5 Fig — The random errors due to the high-speed nanopore sequencing process often appear in a few reads or only in reads from one direction. The presence of variants supported by reads from both directions strongly indicates their existence. (PDF) [file pone.0297231.s012.pdf]

S5 Fig. Evidence of variant existence confirmed by forward and reverse read.

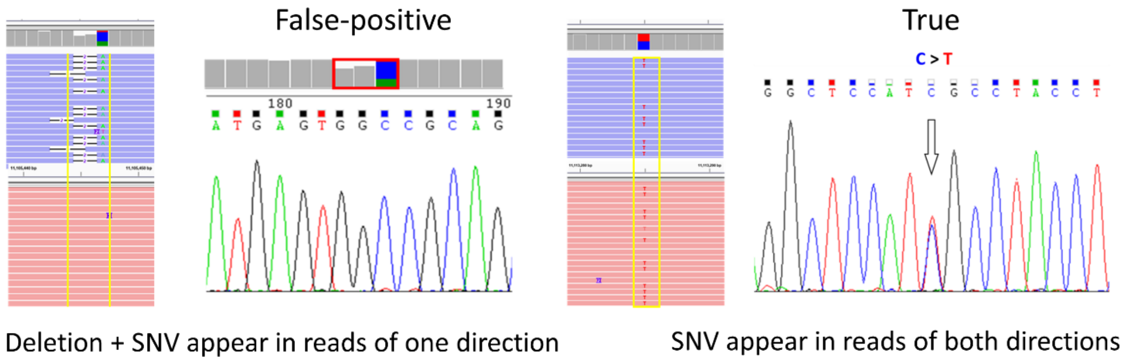

The random errors due to the high-speed nanopore sequencing process often appear in a few reads or only in reads from one direction. The presence of variants supported by reads from both directions strongly indicates their existence.
