# Supplementary material for: CRISPR-Cas9-guided amplification-free genomic diagnosis for familial hypercholesterolemia using nanopore sequencing
Source: PLoS One. 2024 Mar 20;19(3):e0297231. doi: 10.1371/journal.pone.0297231 (PMC10954175; doi:10.1371/journal.pone.0297231)
Supplement: S6 Fig — (PDF) [file pone.0297231.s013.pdf]

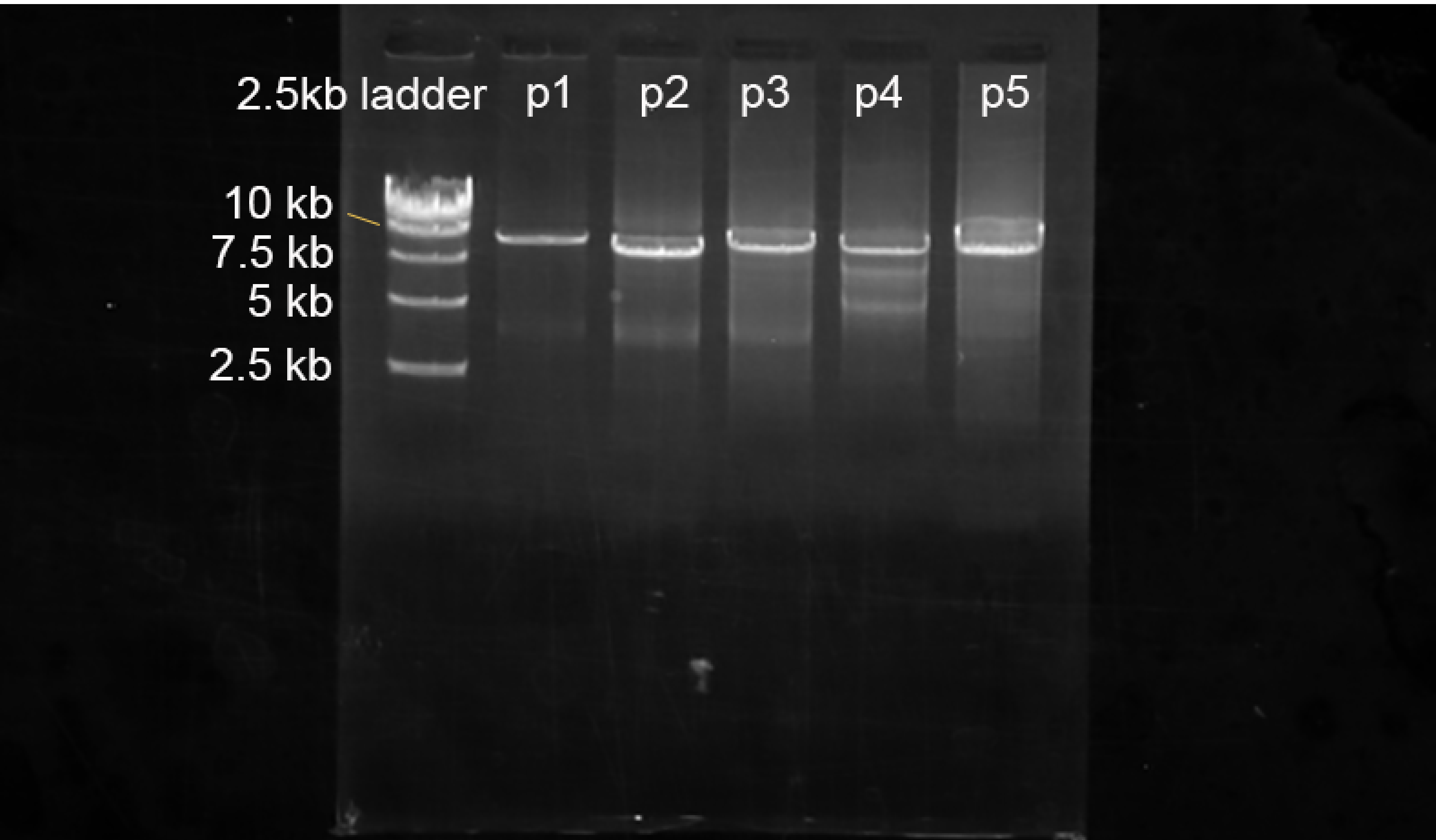

Sample: Control sample
Photograph: 312nm UV(ATTO, Cat #AE-6193)
This image was used in generating **Fig 2B**, illustrating a strategy to amplify the complete *LDLR* using 5 parts in sequence. See **S6 Table** for detailed method.

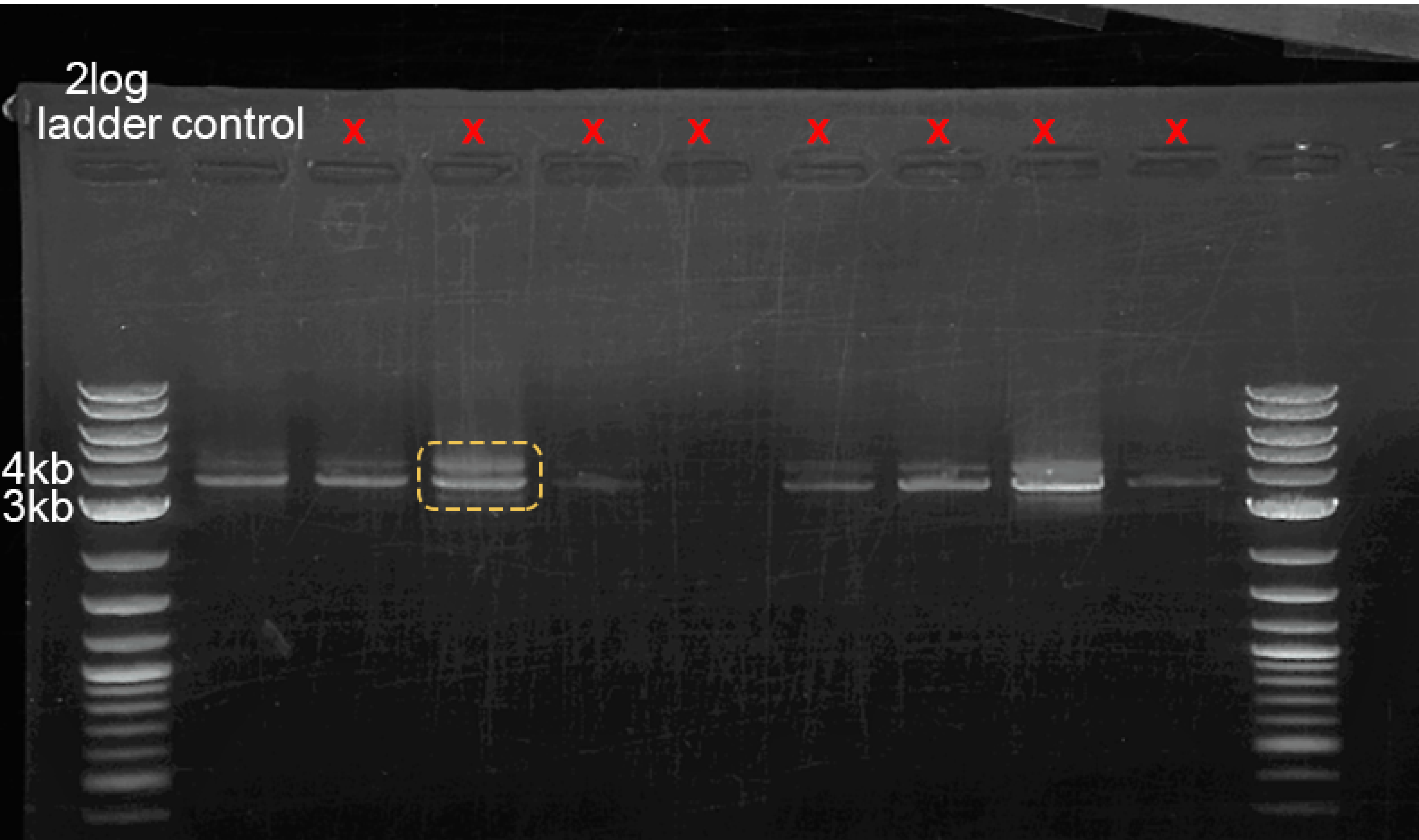

Samples: Control sample + samples exihibiting the false positive deletion (S6 Table conditions) from previous research.
Photograph: 312nm UV (ATTO, CAT #AE-6193)
Note: For clarity, bands within dashed lines are featured for **Fig 2B.** The core conclusions of this study are not impacted.

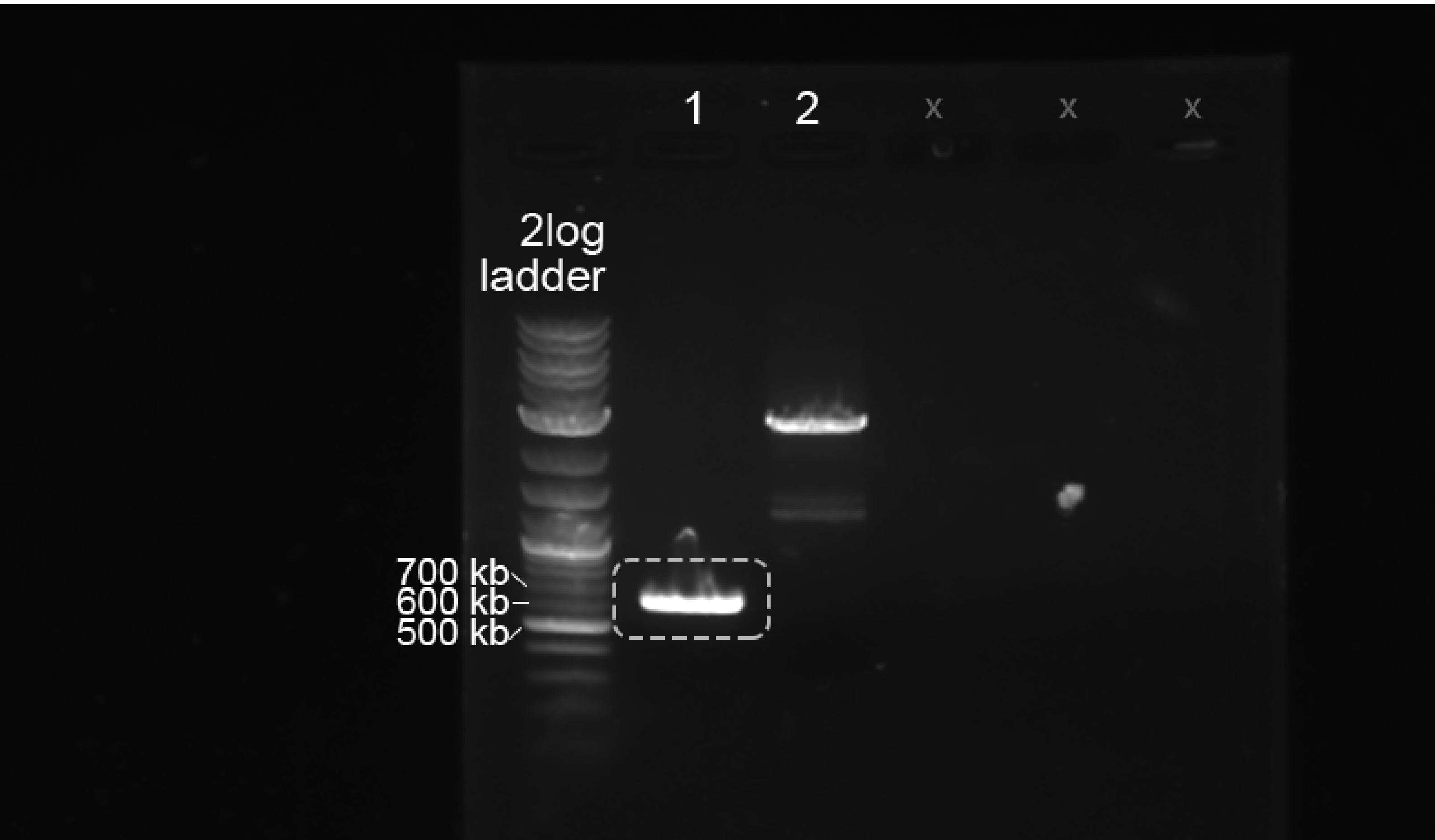

Sample: Individual 4
Photograph: 312nm UV (ATTO, CAT# AE-6193). This image was used to generate Fig 4A. Multiple sets of PCR primers were tested. Lane 1 (with conditons in S7 Table) successfully shows the 624 bp band and confirmed the presence of the deletion, hence the lane 1 band is used.
