# Supplementary material for: CRISPR-Cas9-guided amplification-free genomic diagnosis for familial hypercholesterolemia using nanopore sequencing
Source: PLoS One. 2024 Mar 20;19(3):e0297231. doi: 10.1371/journal.pone.0297231 (PMC10954175; doi:10.1371/journal.pone.0297231)
Supplement: S1 File — (PDF) [file pone.0297231.s014.pdf]

S1 File. Bioinformatic analysis command lines.

1. **Minimap2 for alignment, Samtools for sorting, indexing and filtering ROI bam files**

   cat path/to/fastq > merged.fastq

   minimap2 -ax map-ont --MD -Y reference.fasta merged.fastq | samtools sort -O BAM -o sorted.bam && samtools index sorted.bam

   samtools view -L roi.bed sorted.bam -h -o roi.bam && samtools index roi.bam

2. **Average coverage calculation/finding off-target sites**

   samtools depth sorted.bam > all.cov

   awk '{ total += $3 } END { print total/NR }' all.cov

   survior bincov all.cov 1000 25 > off-target.bed

3. **Samtools picking out full-length aligned reads**

   samtools view -L *gene* [*].innermost.bed roi.bam -h -o *gene*.innermost.bam && samtools index *gene*.innermost.bam && samtools index *gene*.innermost.bam

   samtools view -L *gene*_left_innermost.bed roi.bam -h -o *gene*.left.bam

   samtools view -L *gene*_right_innermost.bed *gene*.left.bam -h -o *gene*.full.bam && samtools index *gene*.full.bam

*. *gene* refers to *LDLR* and *PCSK9*. See table below for genetic location in bed files.

**Name**	**Description**
roi.bed*	chr19:11079432-11143820<br>chr1:55029548-55074853
ldlr.innermost.bed	chr19:11085715-11138029
pcsk9.innermost.bed	chr1:55038317-55064879
*ldlr*_left_innermost.bed**	chr19:11085715-11085730
*ldlr*_right_innermost.bed	chr19:11138015-11138029
*pcsk9*_left_innermost.bed	chr1:55038317-55038327
*pcsk9*_right_innermost.bed	chr1:55064869-55064879

*. 10 kbps flanking the target genes，for better visualization of on-target enrichment performance on IGV.

**. *gene*_left/right_innermost.bed A 10~15 bp region containing innermost crRNA cutting sites.

4. Bcftools for variant calling

bcftools mpileup -Ou -f reference.fasta *gene*.full.bam | bcftools call -mv -Ov -o gene.vcf

5. Annovar for variants annotation

perl convert2annovar.pl -format vcf4 gene.vcf > *gene*.avinput

perl table_annovar.pl --buildver hg38 -remove -polish --csvout -outfile *gene* --operation g,f,f,f,f,f,f -protocol refGene,clinvar_20210123,avsnp150,dbnsfp41a,dbscsnv11,EAS.sites.2015_08,intervar_20180118 *gene*.avinput path/to/annovar_database
